# Supplementary material for: Prevalence and intensity of Schistosoma mansoni infection, and contributing factors in Alamata district of Tigray Region, Northern Ethiopia
Source: PLoS Negl Trop Dis. 2024 Nov 25;18(11):e0012691. doi: 10.1371/journal.pntd.0012691 (PMC11627438; doi:10.1371/journal.pntd.0012691)
Supplement: S1 File — (DOCX) [file pntd.0012691.s001.docx]

1. **Questionnaire to assess socio-demographic, behavioral or environmental factors associated with *Schistoma mansoni* and infection (other parasites) in Alamata district of Tigray Region, Northern Ethiopia.**

**Instruction**: Please fill the blank space for the open ended question and encircle the answer for the given responses of the study participants in the question item.

1. **Socio-demographic and behavioral information**

|  | | | Date ……..…./…….……./…….…. | | | |
| --- | --- | --- | --- | --- | --- | --- |
|  |  |  | Name of data collector……………………….…  Time interview started…………………………. | | | |
|  |  |  | Client code …………………………….…………..…. | |  | |
|  |  |  | **Full Address of participant**  Name of Cluster …………………………….  Name of Kebele:……………………………  Tel no:……………………………………… | | | |
| **1** | **Socio-demographic variables** | | Responses | | | Remark |
|  | Age (in years | | ……………………….……………... | | |  |
|  | Sex | | 1. Male 2. Female | | |  |
|  | Ethnicity | | 1. Tigray 2. Amhara 3. others | | |  |
|  | Religion | | 1. Orthodox 2. Muslim 3. Others | | |  |
|  | Educational Status | | 1. Illiterate 2. Elementary (1-8) 3. Secondary (9-10) 4. High school (11-12) 5. College and above | | |  |
|  | Marital status | | 1. Single/under age 2. Married/co-habited 3. Widowed 4. Divorced | | |  |
|  | Occupation | | 1. Employed 2. Merchant 3. Farmer 4. Housewife 5. Daily laborer 6. Student 7. Unemployed | | |  |
|  | Residence | | 1. Rural 2. Urban | | |  |
| 1. **Behavioral and environmental variables** | | | | | | |
| **2** | | **Behavioral/environmental variables** | | **responses** | | **Remark** |

|  | Do you bath your body in a nearby river/stream or other water body | 1. Yes 2. No |  |
| --- | --- | --- | --- |
|  | Do you swimming or play in nearby river or water body? | 1. Yes 2. No |  |
|  | Do you wash clothes or utensils in a nearby river or water body? | 1. Yes 2. No |  |
|  | Do you work in irrigated agricultural field? | 1. Yes 2. No | If the response to Q.No 2.4 is yes, proceed to Q No 2.5, if No skip to Q.No 2.7. |
|  | Do you make direct contact with water while working on irrigation fields | 1. yes 2. No |  |
| 2.7 | Do you cross rivers or water bodies in your daily activity? | 1. Yes 2. No | If the response to Q.No 2.7 is yes, end your interview here, If the response to Q.no 2.7 is yes, please proceed to Q.no 2.8 |
| 2.8 | How do you cross river or any water body? | 1. Make direct contact with water 2. Wear protective shoes 3. Use bridge to cross |  |

Time completed the interview………………………….

**Thank you!**
