# Supplementary material for: Prevalence and intensity of Schistosoma mansoni infection, and contributing factors in Alamata district of Tigray Region, Northern Ethiopia
Source: PLoS Negl Trop Dis. 2024 Nov 25;18(11):e0012691. doi: 10.1371/journal.pntd.0012691 (PMC11627438; doi:10.1371/journal.pntd.0012691)
Supplement: Attachment — Submitted filename: Response to eviewers.docx [file pntd.0012691.s005.docx]

**One by one response to reviewers**

**Major comments**

**Abstract section**

1. Line 29: Almata changed to Alamata (throughout the text)
2. Line 30: Tigrai to Tigray (throughout the text)
3. Line 35: Inter to inter
4. Line 39: log deleted
5. Line 44: Significant gender difference🡺deleted
6. Line 46: among males and 18.6% (175/941) among females (χ^2^= 10.146, P-value=0.001) 🡺deleted.

Amendment (current line: males accounting for 26% 26% (204/821) of the infection

7. Line 47:group 10-14 years🡺deleted

Amendment: groups of 15-19…

- The feedback provided in the abstract section has been carefully considered and incorporated into the revised manuscript. We have made the necessary amendments to ensure that the abstract accurately reflects the key findings and objectives of the study. Table 5 is also amended accordingly.

Line 57 -Line 73 (in the revised form): Author summary inserted

**Background**

1. Line 89:NTD🡺Neglected tropical diseases
2. The comments from the reviewers “*The background is brief. I suggest to revise it by incorporating relevant details including: historical data on the prevalence of S. mansoni in the district, overview of existing schistosomiasis control and preventive strategies in the district and nationally, explanation of gaps in knowledge that the study aims to address and justification for the need for updated data. The rationale, as it appears currently, is not convincing to carry out the study*.

🡺The comment is valid and significant; it provides important insights that can improve the paper's overall quality. We have included a thorough history and a summary of Ethiopia's schistosomiasis control efforts in response to your input. We have also incorporated earlier data on the parasite's prevalence in the research area. This strengthens our discussion and also provides a clearer understanding of the challenges and progress made in combating schistosomiasis in the region. Besides, valid justification is made to the significance of the study.

🡺Hence, the changes are shown from line 92-130 of the old version or ***90-122*** of the revised form

**Methods**

1. **Regarding description of study setting**

The feedback provided is indeed valid, and we appreciate the insights shared. In response, we have added detailed information about the study area to provide a more comprehensive context for our research. This includes an overview of the socioeconomic activities prevalent in the region. We have also highlighted the primary sources of drinking water, information about the presence of nearby rivers, which are crucial factors influencing both the local economy and the risk of schistosomiasis transmission. By incorporating these details, we aim to enrich the reader's understanding of the environmental and social dynamics that impact the study area. Besides, ***map of the study area*** is shown (generated) in ***Figure 1*** as per the suggestion of the reviewers and is provided as a separate file.

🡺The changes are shown line 125-147 in the revised form 133-164

2. Lines 96-97 (old version): Description of the study period is made clear and shown line **149-152** of the revised form.
3. Inclusion/exclusion criteria: An Eligibility criterion is inserted shown line 170-172.
4. Line 99 (old version): regarding **Sample size formula:** the equation function is used and each variable within the equation is defined as shown in line 157-164 of the revised form. : The equation function was utilized to calculate the sample size in response to the valuable comments provided. Besides, the various parameters such as expected prevalence, the desired confidence level, and the margin of error and others are well described.
5. Line 103-109 (old version): The sampling procedure is amended and made clear as shown in line 165-178 in the revised version.

***Regarding the term cluster***:

The term "cluster" refers to a local administrative unit used in the health sector in the study area. In this context, various kebeles are grouped into larger entities called clusters to facilitate management. Each cluster typically includes one central health center and two or more health posts that provide services to the population within its catchment area, which encompasses two to four surrounding kebeles. Basically, a cluster is larger than a kebele and can consist of two or more kebeles, depending on local administrative structures. So, we took the term cluster which is used by local health administration.

***Selection of clusters:***

We would like to inform you that after careful consideration, we randomly selected five clusters from six available, and these clusters have been utilized by the local administration (particularly the health sector in the district). So, this random selection ensured a representative sample that minimizes selection bias and allows for robust exploratory analysis. This approach enhanced our resource efficiency, enabling us to focus on a manageable number of clusters while still capturing diverse insights. We believe that selecting five was sufficient to effectively represent the study area. It also provided flexibility in our analysis, enabling us to explore different cluster combinations and their impacts without preconceived biases.

**Regarding sampling**:

Based on the information from local administrators, the total population of the Raya Alamata (rural Almata was) estimated at 85, 403. Following this, we got the estimated population of each cluster and the sample size for each cluster was calculated based on proportional allocation as follows (except for Alamata town which will be discussed later in this section):

a. Waja cluster: According to the local administrators, the population of the cluster was around 39048.

(38982/85403) *1765҄==807,

b. Tumuga cluster: estimated population= 17293

(17293/85403) *1768=358 selected for the study

c. Selenwuha cluster: There were an estimated population of 17293, and hence

(8405/85403) *1765=174 participants selected

d. Garjale Cluster: Estimated population=24, 454

(14443/85403) *1765= 299 selected in the study.

e. Alamata town: The reports from the woreda health bureau and the reports from the Woreda administration (political administration) about the population of the town greatly varied. So the research team discussed on how to allocate the sample to this specific area. It is well known that *S.mansoni* is more prevalent in rural populations compared to urban settings. The WHO also recommends that schistosomiasis control programs prioritize areas with the highest infection rates. Hence, taking large samples from rural areas aligns with these guidelines as it ensures that the study captures most pertinent data for understanding the epidemiology of *S.mansoni* in the study area. Different studies have also evidenced that schistosomiasis, particularly *S.mansoni* (in our case), is more prevalent in rural areas where water contact activities such as as farming are very common.

Besides, while deciding, we had to assure that it was both statistically and epidemiologically sound. As it has been shown by different studies (Savioli et al, 2006 and Cohen et al, 1983), sampling representative sample (as low as 5-10%) of a population can provide sufficient data (reliable estimates of prevalence and risk factors) when appropriately stratified. Hence, taking these considerations in to account, we decided to allocate the sample based on proportional allocation for the other rural clusters (as shown above: a-d) and to allocate the rest of the sample to Alamata town which was 7% of the total sample size i.e 127. Since, we did not have exact number of people in the town, we randomly selected two kebeles (out of the 4) from the town, and we almost equally distributed the sample to each of these kebeles. So, we systematically selected the participants by selecting every other household in a row in the town till we get the desired sample size from each kebele.

6. **Line 116-119:** regarding questionnaire revised as shown line **181-188** of the revised version. Besides, the questionnaire is submitted as additional file.
7. **Line 123-**124: Revised as shown in line **190-206 of the revised form.**
8. **Ethics statement**: reference number of IRB indicated. Besides, results have been communicated to the local health authorities for initiation of treatments.
9. **Line 247-257 (new):** a new section (operational definition) was inserted in to the revised form which was not in the old version to make clear the differences between cluster, kebele, and ketena.

**Results**

10. **Line 175:** comments are valid and revised accordingly as shown in line **260-265**
11. Line 181-184: Figure 2 has been deleted and the information is incorporated to table 1. Besides, Figure 2 in the revised form refers to another figure shown sampling procedure. Because one additional figure showing map of the study has been generated as Figure 1.
12. **Line190-191 and regarding Table 2:** significant revision has been made to Table 2 as shown 267-269
13. **Lines 199-205:** calculation of Prevalence in each cluster is amended as shown line 293-299 of the revised form. Besides, the contents and labels of Figure 3 are amended (Figure 3 attached separately).
14. **Regarding use of Arithmetic Mean (Line 210)**

We used arithmetic mean (AM) to calculate the intensity of infection in this study as shown in Line 300 of the revised form.

***Justifications for using AM:*** Although our data was not normally distributed, we preferred to report the intensity of infection using arithmetic mean (AM) rather than geometric mean (GM) for the following reasons.

I. The GM underestimates the true mean as it reduces the effects of outliers in the sample. Particularly when we compare group intensities of infection, GM reduces the influence of the high values which typically occurs in children so that the peak infection intensity would not be indicated in the age group where the participants are carrying the heaviest infection levels. So as the GM hides, extreme values we used the AM.
II. The arithmetic mean, on the other hand, is sensitive to changes in all data points, which can be useful when assessing overall infection intensity. It captures the impact of high-intensity infections, which might be diluted in geometric calculations. eg: a small proportion of individuals disproportionately carrying an intensity of infection are better captured by AM.
III. The intensity of infection did not exhibit normal distribution even after it was log transformed. Hence, we decided to use the AM in a way that it can show a more accurate representation of the central tendency.
IV. The arithmetic mean is easier to interpret and understand, making it more accessible for non-statistical audiences in clinical or public health contexts as it can show the overall burden of disease within a population.
V. The arithmetic mean aligns with other epidemiological metrics commonly used in health assessments, facilitating comparisons across different studies and populations. Overall, while the geometric mean has its uses, the arithmetic mean offers clearer insights and practical implications for addressing *S.mansoni* infection intensity.
15. Line 215-217: revisions have been incorporated as shown line 300-308 of the revised form.
16. ***Why intestinal parasitic infections (STH) were not included in the study?***

Although the purpose of the study was to investigate the prevalence of *S.mansoni* and associated risk factors, we also investigate the kato slides for the presence of other parasites’ ova, larva or adult. However, we exclude the results from this manuscript for the following reasons:

a. This research is part of a PhD project which deals about Liver disease and risk factors in the specific area where *S. mansoni* is considered one of the risk factors for liver disease among others. In the large project, factors such as HBV*, S.mansoni*, Alcohol, consumption of khat, and other factors are studied singly and in combination.
b. The primary objective of this study was to investigate the prevalence of *S.mansoni* and associated risk factors. Hence, including STH in the manuscript would divert attention from the specific aims of our research and could dilute the findings related to *S. mansoni*.
c. By concentrating solely on *S.mansoni*, we believe that our study can provide a more in-depth analysis of its specific epidemiology and associated risk behaviors. It allows for a more streamlined discussion and interpretation of results, making it easier for readers to understand the implications of our findings. Besides, by narrowing the focus, it can provide targeted recommendations and insights specifically related to *S. mansoni*, which may be more actionable for public health interventions.

Hence, for the above reasons we excluded STH findings from the manuscript. However, the data can be used for local consumption (prevention and control measures in the area) i.e. the results will be communicated to the local health sectors. In the mean time, we have attached the data about S.mansoni and other STHs in the excel file labeled as S1 data in supporting information.

**Discussion**

1. Line 263: comments to mention the interventions undertaken🡺

While there is no published data on interventions specifically carried out in the study area, Ethiopia has been implementing targeted deworming programs aimed at controlling intestinal parasitic infections among school-age children to decrease morbidity and mortality. Therefore, as part of these deworming initiatives and given the prevalence of *S.mansoni*, it is widely believed that control measures using mass drug administration (MDA) have been undertaken. Nonetheless, we have included data regarding the control efforts conducted in Ethiopia as shown in lines **375-381** of the revised form.

2. 265-266:corrected 🡺line 381
3. 280🡺 current control measures🡺 line 375-381
4. Line 308: prevalence among males and females🡺 comments are valid and omitted from the manuscript. Line 421…………..
5. **Conclusion** (line 329 of old version: Comments well taken and problem well addressed as shown in lines 441-452.

**References**

Efforts have been made incorporate to replace the references with updated references which were published with the last 10 years. However, we also included some references older than 10 years to show the control history of Schistosomiasis in Ethiopia and also other books and methodological references such as (to show kato-katz techniques and cut point of intensity of infection) and to show other pertinent scientific justifications.

**Minor comments**

- All other minor commented have been addressed and incorporated **in the new revised form of the manuscript** (clean-copy) and are also shown using track changes in the Revised with track changes (marked-copy) form for the interest of the reviewers.
