# Supplementary material for: Prevalence and intensity of Schistosoma mansoni infection, and contributing factors in Alamata district of Tigray Region, Northern Ethiopia
Source: PLoS Negl Trop Dis. 2024 Nov 25;18(11):e0012691. doi: 10.1371/journal.pntd.0012691 (PMC11627438; doi:10.1371/journal.pntd.0012691)
Supplement: Attachment — Submitted filename: Reviewer comments.docx [file pntd.0012691.s004.docx]

**Reviewer’s report**

**Date: 21 July 2024**

**Tittle: Prevalence and intensity of Schistosoma mansoni infection, and contributing factors in Alamata district of Tigray Region, Northern Ethiopia**

This study determined the prevalence of *S. mansoni* among inhabitants in Alamata district of Tigray Region in Ethiopia. Over 1700 participants from various clusters within the district were enrolled. Data on factors associated with *S. mansoni* were collected through questionnaires, and duplicate Kato-Katz thick smears were processed to detect and quantify *S. mansoni* in stool specimens. The findings of the study have important implications for schistosomiasis prevention and control efforts in the district. However, analysis and presentation of the results require extensive revision. Moreover, the manuscript needs major revision for language and grammar to be considered for publication. The following are my specific comments on the manuscript.

**Major comments**

**Abstract**

Line 43: Please check and correct 26% to 24.8%. Note: “significant gender difference” contradicts the results of multivariable analysis (Table 5). I suggest to present findings of the multivariable analysis.

**Introduction**

The background is brief. I suggest to revise it by incorporating relevant details including: historical data on the prevalence of *S. mansoni* in the district, overview of existing schistosomiasis control and preventive strategies in the district and nationally, explanation of gaps in knowledge that the study aims to address and justification for the need for updated data. The rationale, as it appears currently, is not convincing to carry out the study.

**Methods**

1. Description of the study setting needs clarity, and important details are missing: line 84-85 “The study was conducted in Alamata district…. Alamata district is divided into rural and urban woredas (districts)”. This is confusing. If woreda and district be used interchangeably, I suggest to use either them. The same is true for cluster/kebele (line 86) and ketene (line 105). Are these three the same or different? Authors should follow the actual local administrative hierarchy and naming to clarify it. Description of the study area also needs some details eg. livelihood of the people in the district (socio-economic activities), major source of drinking water in the district, presence of fishing activity and/or irrigation scheme in the. I also suggest to have map of the district indicating the sampled “clusters” and relevant features (eg. line 91) of the study area in the manuscript.
2. Lines 96-97: Description of the study period is not clear. The “study period” and “data collection period” appear different.
3. Line 99: Use the equation function to insert the formula to render it more mathematically fit. Also, define what each variable within the equation represent.
4. Line 103: The sampling procedure requires revision to clarify it. From the prior description of the study setting, it appears that there are a total of six “clusters” in the district - five rural and one urban (Alamata town). Five clusters out of the six were randomly selected. Did you have any reason to go for five instead of all the six clusters. Were the "clusters" used by local administration or made for the purpose of the study?
5. Line 109: How you actually sampled the individuals from the households need to be mentioned. How did you allocate the mentioned number of individuals to each kebele? (eg 127 individuals from kebele 01 and 03) and so on (Fig 1). Furthermore, what proportion of individuals was enrolled from Kebele 01 and 03 is not indicated.
6. Inclusion/exclusion criteria of the study participants in not mentioned at all.
7. Line 116-119 (Questionnaire survey): Important details are missing: eg. How was the questionnaire prepared, in what language, if translated to local language (what was the local language), who interviewed the study participants (qualification to do the interview), training on the tools… Also, a mention to what socio-demographic and environmental data were included in the questionnaire is required. The questionnaire may also be included as additional file and be submitted.
8. Line 123-124: “ stool samples ….transported to nearby temporary center” is not clear. Do you mean health center. More clarity on the field collection/field set up is required. Mention whether you collected the stool samples house-to-house or you gathered the selected study participants to a common place. In what condition did you transport the stool samples? Also, indicate where you carried out the Kato-Katz procedure.
9. Ethics statement: Line 135- Reference number of the IRB letter be included. What did the research team do for participants who became positive for any intestinal parasites, including *S. mansoni*?

**Results**

1. Line 175: “The majority …. recruited from Waja”. This is expected, as the majority of samples are allocated to this cluster. You may incorporate the information of Fig 2 into Table 1 and deleted the figure. Line 177 “….45.3% were single…” actually they are a combination of single and under age (see table 1).
2. Lines 190-191: Description of the denominator is not correct. Revise it according to the information in Table 2
3. Table 2: The variable “Clusters” be also included in the table. How the percentage of intensity of infection is calculated is misleading. I suggest the denominator be total positive of each group. For instance, the sum of the percentage of light, moderate and heavy infection intensity among males should be 100. This be calculated similarly for the remaining responses of the variables. Note: there is no need to repeat the range of egg count, as it was already defined under the methods (write only light, moderate and heavy for the heading). For the “Educational status”, the “illiterate” group apparently also included underage children and this should be indicated.
4. Lines 199-205: The statements are misleading. While calculating the prevalence of *S. mansoni* in each cluster, the denominator should be total number of study participants in each cluster, not the total positive. Fig 3 should also be revised accordingly. Y-axis label of Fig 3 be added.
5. Line 210: Authors need to specify whether arithmetic mean or geometric mean was used to calculate the mean. As parasite counts often have skewed distribution, the geometric mean is recommended, to account for outliers.
6. Line 215: Refer to the data showing infection intensity for the description. Note that this statement should also be revised in line with the comment on infection intensity in Table 2.
7. Lines 221-227: Revise the paragraph to improve quality of the written English.
8. Table 4. It is not clear what “Frequency” stands for. Also, the “Districts” were referred to as “Clusters” under the study area. Consistency is needed.
9. Surprisingly, intestinal parasitic infections other than *S. mansoni* were not reported in this study. Soil transmitted helminths should be detected and intensity determined by the technique utilized in the study. There is no mention of STHs at all in the manuscript. Either data of the STHs should be included or justification for the missing this relevant data be presented.

**Discussion**

1. Line 263: “Despite great efforts made in the fight against schistosomiasis….” Worth specifically mentioning the interventions undertaken in the district, and discuss.
2. Line 265-266: “…21.5% indicating that the parasite continued to be a major public health problem in the country” Be cautious upon generalization of your finding to the country.
3. Line 280: “…current control measures…” Please specify and discuss the current/past control measures undertaken in the area.
4. Line 308: your finding shows similar prevalence of *S. mansoni* infection among males and females, justifying the reason for high prevalence among males contradicts this.
5. The conclusion part is quite brief, comprising only a single sentence (the remaining being recommendations). I suggest to expand the conclusion part summarizing your key findings. The recommendations should be aligned with your conclusion and major findings.
6. References – To ensure the study is based on most recent data and developments in the field, I suggest to replace references older than 10 years with up-to-date ones (with the exception of books or standard methodological references or foundational studies).

**Minor essential comments**

- Line 30: delete “design”, Line 38: delete “log”
- “region” when it refers to Tigray Region, as in the title, should be written as “Region” throughout the manuscript eg. Lines 80, 84, 96 and so on.
- I suggest correcting the spelling of the words: “Burueaue” to “Bureau” (line 135), “Result” to “Results” (line 173), “(N=…) to “(n=…) in table/figure captions.
- Also, two versions of the following words are found in the manuscript and be corrected: “Tigrai” and “Tigray”, “Almata” and “Alamata”, “kebele” and “kebelle”
- Line 69: “NTDs” is used only once. As such I suggest to define it without the abbreviation
- Define what “*Kebele*” is in the local area (line 86)
- Line 86: define or replace “a.k.a”
- Line 123: How were study participants actually communicated to bring “approximately 3g” of fecal sample.
- Line 149: rephrase the sentence “I closely followed and led….”
- Table 2: S. mansoni be written in italics as *S. mansoni*, similar correction should also be made at line 59
- Table 4: Something must have crept in the variable “Educational status”. Also delete “Total”
- Tabel 5: the variable “Schistosoma infection, n (row %)” delete the word “row”
- Line 211: “egp” is already defined under the methods section upon initial use, no need to define it here.
- Lines 212 and 213: The egg count range for *S. mansoni* infection intensity classification as light, moderate and heavy is already stated under the methods part, no need to repeat it.
- Line 241: subtitle better be written as "Factors associated with *S. mansoni* infection"
- Lines 245-246: Delete “… with P-value less than 0.025 in the bivariate analysis were considered …*S. mansoni* infection”, as this is already indicated under Data analysis. BTW, the cutoff p-value indicated under the Data analysis is 0.2, not 0.25.
- “multivariate” be written as “multivariable” throughout the manuscript
- Line 320: use uniform citation style.
- Lines 338 and 339: The reason(s) for acknowledging the institutions be indicated.
